# Lived Experience Engagement in Service Design and Improvement: Investigating Meaning and Impact

**DOI:** 10.1177/15394492251396021

**Published:** 2025-12-13

**Authors:** Yuk Ying Sophia Wong, Carolynne White, Aislinn Lalor, Katie Larsen, Ellie Fossey

**Affiliations:** 1 Monash University, Frankston, Victoria, Australia; 2 Mind Australia, Burnley, Victoria, Australia

**Keywords:** mental health, occupational engagement, qualitative research, participation

## Abstract

Involving people with lived experience is important to mental health service design, development, and research. This study explored the personal meaning and impact of participating in lived experience opportunities for consumers and carers. Six semi-structured interviews with consumer and carer volunteers at an Australian community mental health service were analyzed through an interpretative phenomenological lens. A steering group, formed by staff with lived expertise, guided the research. Five themes were identified: “Transforming negative experiences into positive contributions,” “Self-growth through lived experience participation,” “Connections and support through lived experience participation,” “Considerations for supporting consumers and carers in a team,” and “Considerations for setting up voluntary lived experience participation roles.” Lived experience engagement is meaningful to consumers and carers. Creating safe-enough environments that support participation in decision-making requires valuing diverse opinions, having supportive facilitators, acknowledging demands of sharing lived experiences, and paid remuneration.

## Plain Language Summary

Meaning and Impact of Lived Experience Engagement

Involving people with lived experience of mental health challenges and recovery (consumers) and their supporters (carers) is important for mental health service design, development, and research. This study explored consumers’ and carers’ experiences of participating in lived experience opportunities, including the meanings and impacts. This paper explains the learnings from three consumers and three carers who volunteer at an Australian community mental health service. The interviews were analyzed through interpretative (understanding) phenomenological (experiences) analysis, which explores how people understand their experiences. Staff with lived expertise formed a steering group, which guided the research. The identified themes include: (a) Transforming negative experiences into positive contributions. (b) Self-growth through lived experience participation. (c) Connections and support through lived experience participation. (d) Considerations for supporting consumers and carers in a team. (e) Considerations for setting up voluntary lived experience roles. Involvement in mental health services is important for consumers and carers. Key strategies for creating safe-enough environments include valuing diverse opinions, having supportive facilitators, recognizing challenges of sharing, and providing payment.

## Introduction

There is a growing recognition of the role of people with lived experience in the mental health sector. Internationally, the World Health Organization (WHO) has called for the meaningful engagement of people with lived experience of mental health challenges at all levels to strengthen mental health systems and improve outcomes for those who use services and are directly impacted by policies and programs (WHO, 2023). In Australia, the Royal Commission into Victoria’s Mental Health System (2021) emphasized that inclusion and leadership of people with lived experience would be the foundation for the future mental health system. Involving people in decisions that impact their lives is not just a moral imperative but a human right that challenges traditional power imbalances and fosters the design of more relevant, cost-effective and accessible services that improve outcomes (Sartor, 2023; WHO, 2023). However, to realize these benefits, lived experience participation must be authentic and should involve intentional efforts to create a safe-enough environment to ensure psychological safety (Reynolds, 2012).

The perspectives and input of both consumers who have “personal experience of mental health challenges”; and families, carers and supporters with lived experience of “supporting a person living with mental ill-health or psychological distress” are valued in the mental health sector (Department of Health, 2024, para. 4). For this article, the terms consumers and carers are used as they were the terms preferred by participants in this study and align with terminology used in the lived experience sector (Department of Health, 2024).

A range of lived experience opportunities exist across research, service design, service delivery, governance, policy planning and development, and education (Department of Health, 2021; WHO, 2023). Such opportunities include the formal lived experience workforce with designated roles (e.g., peer support workers, policy advisors, and researchers), and partnerships with consumers and carers on a voluntary basis, with or without remuneration (Department of Health, 2021; Sartor, 2023). This study focuses on voluntary lived experience opportunities relating to service user engagement in high-level service planning, with participation spanning three levels: consulting, involving, and collaborating (International Association for Public Participation, 2024).

Despite growing recognition of the value of lived experience participation, consumers and carers continue to navigate challenges within these opportunities. Studies indicate that consumers often feel their honest contributions are unwelcome and encounter prejudicial beliefs from health professionals (Ehrlich et al., 2020). In addition, research highlighted that tokenism in mental health services undermines the effectiveness of participation opportunities, leaving consumers feeling confused and undervalued. For example, using medical jargon in important conversations may exclude people (Gee et al., 2016; Lammers & Happell, 2003). Similarly, carers engaging in lived experience work often found their needs were underappreciated, and their voices were disregarded (Bradley, 2015).

Despite these challenges, consumers found their participation to be enriching (Bennetts et al., 2013). This duality highlights the importance of meaning-making as an intrinsic element in understanding human experiences (Fossey et al., 2016). Consumers reported different reasons for participation, including a sense of purpose and a deeper understanding of their mental health and identity (Lammers & Happell, 2003). Consumers were also driven by making a difference to those accessing mental health services (Hawgood et al., 2022). *Consumer and carer engagement: a practical guide* was co-produced by consumers and carers and highlighted that through lived experience opportunities, consumers and carers can better influence system change and have their voices heard (National Mental Health Commission, 2019).

The literature outlined above offers some insights into the experiences of consumers and carers who engage in service provision, education, and research opportunities. However, understanding engagement from the perspectives of those in voluntary lived experience roles remains limited, especially for carers. The duality of lived experience participation, marked by both meanings and challenges, invites further exploration to better interpret and contextualize people’s experiences. Therefore, this qualitative study aimed to explore the personal meaning and impact of lived experience engagement in mental health service design and development for consumers and carers. This study is grounded in the doing, being, becoming, and belonging framework (Wilcock, 1998, 2006), which highlights the dimensions of meaning in lived experience participation as an occupation, and provides insights into occupational therapists’ roles in facilitating safe and meaningful engagement.

## Method

This qualitative study employed interpretative phenomenological analysis (IPA) to allow an in-depth exploration of the essence and meaning of the subjective experience of lived experience engagement, seeking deeper meaning beyond the text (Creswell, 2013; Harper, 2011). IPA involves a double hermeneutic process, in which researchers make sense of how participants make sense of their experiences (Smith & Nizza, 2021). Reflexivity is therefore essential in IPA, requiring researchers to critically reflect on their own positionality and interpretative influence throughout the research process (Alase, 2017).

This study was guided by a Lived Expertise Steering Group, which consisted of staff at Mind Australia, who brought consumer or carer lived expertise based on their collective understanding of the lived experience movement (Byrne & Wykes, 2020). Collaboration with the Lived Expertise Steering Group fostered connection and partnership between researchers and stakeholders (Vaughn & Jacquez, 2020). The Lived Expertise Steering Group informed the research design and development of participants’ materials as well as interpretation of themes and their implications (refer to Table 1). This study was approved by Monash University Human Research Ethics Committee (Project ID: 36065).

**Table 1.** Lived Expertise Steering Group Schedule.

1. Initial meeting	Review and refine research aim Review and refine research questions Review and refine research design Develop the interview guide Develop the demographic survey
2. Prior to ethics application	Review and refine participant consent forms Review and refine participant explanatory statements
3. Prior to data collection	Prepare for and trial the interview
4. After data collection	Discuss initial themes and potential implications

### Participants

This study was conducted in collaboration with Mind Australia, a national, community-managed mental health service provider (Mind Australia, 2025). In 2020, Mind Australia established the Lived Experience Advisory Team (LEAT), which consisted of consumer and carer representatives from diverse backgrounds. LEAT members engage in opportunities related to strategic decisions, service co-design, and business development.

LEAT members were invited to participate in this study based on the following criteria. Inclusion criteria: (a) identify as a consumer or a carer, (b) a current member of the LEAT. Exclusion criteria: (a) having a single participation opportunity, (b) no lived experience engagements since 2019, and (c) unable to give consent due to current mental health challenges.

#### Recruitment

Prior to data collection, the student researcher introduced herself and the study’s aim to LEAT members at a quarterly meeting. After the meeting, invitations to participate, a consent form and demographic survey, were sent to all members through a Mind Australia staff member. Interested individuals contacted the researcher via email, who arranged a short phone call with potential participants to discuss the project further. Once the demographic survey and signed consent form were received, the researcher scheduled interviews with participants. To ensure accessibility and support safe-enough engagement, all participant materials were reviewed by the Lived Expertise Steering Group for clarity and appropriateness. A list of support services was also included in the participant materials. Participants had multiple opportunities to seek clarification and led the interview scheduling process.

Seven individuals expressed interest. Six returned their consent forms and demographic surveys and completed the interviews. The remaining participant chose not to participate due to scheduling conflicts. This sample size (*n* = 6) is suitable as IPA studies emphasize quality of data for rich analyses, over quantity (Larkin & Thompson, 2011).

### Data Collection

The student researcher conducted semi-structured interviews from March to April 2023, using an interview guide developed in collaboration with the Lived Expertise Steering Group. The guide included eight open-ended questions to gather in-depth insights (Creswell, 2014). Interviews were conducted via video conferencing software and were transcribed verbatim using participants’ chosen pseudonyms. The de-identified transcripts were returned to participants for review. All identifying materials were stored on a password-protected drive and will be deleted in five years. An audit trail was maintained to document the raw data and each step of the analytical process.

#### Remuneration

Participants were paid AUD$35 in recognition of their time and expertise, following Mind Australia’s paid participation procedure.

### Data Analysis

Interview data were analyzed using IPA to explore participants’ personal experiences and the meanings of these experiences (Larkin & Thompson, 2011). The IPA process described by Smith and Nizza (2021) was followed: (a) immersing in data and writing exploratory notes; (b) developing preliminary themes from the first transcript; (c) clustering preliminary themes and creating a coherent table; (d) analyzing subsequent transcripts; (e) connecting themes across cases to construct a final table of superordinate themes; and (f) writing up the final themes.

In IPA, acknowledging positionality is essential as the researcher’s experiences and beliefs may have influenced the interpretation of participants’ narratives (Alase, 2017). As the primary researcher, I was a student learning what it means to be an occupational therapist, who is in a profession that values people’s stories and recognizes their environmental context. This positionality informed attentiveness to participants’ narratives and reflections on the roles of staff and facilitators. I engaged in reflexivity by actively using a journal to record impressions, interpretations, and self-reflections. Regular discussions with co-authors also ensured interpretations were grounded in participants’ experiences while acknowledging each researcher’s lens.

Two co-authors (K.L. and C.W.) work from lived expertise perspectives, grounding their work through the lenses of consumer and carer, respectively.

#### Dissemination

A written summary of the research findings was emailed to all LEAT members and shared with Mind Australia.

## Results

Six LEAT members participated in this study, including three consumers and three carers. To protect anonymity, demographic characteristics are presented collectively. The study included consumers and carers aged 20 to 70, with diverse identities such as being culturally and linguistically diverse; lesbian, gay, bisexual, transgender, intersex, queer; or person living with a disability. Participants resided in different states of Australia, including rural and regional areas, and had engaged in lived experience opportunities for over 2 years. Interviews lasted 35 to 65 minutes, averaging 49 minutes. Five overarching themes were produced from the interviews and were further categorized into sub-themes (refer to Table 2).

**Table 2.** Qualitative Analysis: Identified Themes and Sub-themes.

Themes	Sub-themes
Transforming negative experiences into positive contributions	Opportunities for feedback Satisfaction when being acknowledged Lived experience is valuable to clinicians
Self-growth through lived experience participation	Provides structure and reflects recovery journey Fosters self-development and career advancements for consumers
Connections and support through lived experience participation	Profound understanding from others with similar lived experiences Carers felt understood and supported through engagement
Considerations for supporting consumers and carers in a team	Importance of having diverse voices Diverse views on working with both consumers and carers Qualities of supportive facilitators
Considerations for setting up voluntary lived experience participation roles	Cost and constraints of lived experience participation A need for increased accessibility Differing attitudes on paid roles

### Transforming Negative Experiences Into Positive Contributions

#### Opportunities for Feedback

The majority of participants had experienced barriers to accessing services as a consumer or carer. Through their lived experiences, participants gained insights into the faults of the mental health system along with opportunities for improvement. Participants appreciated having an avenue to share their wisdom and facilitate necessary changes.

Lenny (consumer), Charlie (consumer), and Julia (carer) were motivated to influence other people’s personal or relational recovery journeys, respectively. They co-facilitated training, created marketing and practice materials, and were on interview panels. Lenny (consumer) stated that if their lived experience “can help either a carer or a consumer,” they will continue sharing it. Charlie (consumer) believed that consumers and carers’ contributions could “change something we really need” and benefit others in the system, and Julia (carer) hoped their learnings could advise organizations as to “how they’re performing.”

Similarly, for Jamie (carer) and Jordan (carer), if sharing their struggles helped others, their negative experiences could now be associated with positive value. Seeing others benefit, especially loved ones who were still in the mental health system, was important:

When it’s your loved one, and they’re finding life very difficult, it’s just this instinct. . . you want to see them helped and functioning to the best of their ability. It does become a passion. (Jordan, carer)

#### Satisfaction When Being Acknowledged

Almost all participants expressed a feeling of satisfaction and validation when their contributions were acknowledged. For example, when Charlie’s (consumer) contribution was recognized by others, they felt “wanted” and “needed.” Jordan (carer) described being in a group where others are “hungry for your information” as a “complete turnaround” from past experiences in life. They found it “rewarding” to have their voice respected and appreciated rather than disregarded.

#### Lived Experience Is Valuable to Clinicians

Three participants emphasized how health professionals should consider lived experience perspectives. Vicki (consumer) shared that they had experienced bias from practitioners and believed clinicians could benefit from learning about the consumer movement.

Jordan (carer) and Jamie (carer) also believed their perspectives and experiences were invaluable, and clinicians often didn’t see the whole picture as they did:

So lived experiences are the nitty gritty and the boring batshit. Twenty-four hours a day, what goes on, and how to handle that. With all due respect, the clinicians, they don’t see that, and they don’t see any of the things that flow from that. (Jamie, carer)

### Self-Growth Through Lived Experience Participation

#### Provides Structure and Reflects Recovery Journey

Involvement in lived experience opportunities provided routines and structures for participants. Lenny (consumer) appreciated having “more structure’ and “routine” back in their life after being unwell as a consumer. Likewise, Jamie (carer) described their involvement as a “meaningful” way to “occupy” their time.

For Vicki (consumer), their performance in lived experience roles reflected their mental state. If they struggled to keep up with the demands of the role, it was a sign their mental health needed extra attention:

If I wasn’t involved in something. There wouldn’t be accountability, there wouldn’t be feedback. (Vicki, consumer)

#### Fosters Self-Development and Career Advancements for Consumers

All consumers in this study experienced self-development and career advancement through lived experience participation. Consumers described facing many challenges in building skills and finding suitable paid work. Vicki (consumer) shared their struggle in obtaining employment and said it was “incredibly hard to get work.”

However, Vicki stated that utilizing their lived experience for something productive helps break “the cycle of constantly being unemployed or underemployed.” It expanded upon not only their skills, but also built their professional profile. Lenny (consumer) and Charlie (consumer) also found that their lived experiences and past engagements gave them confidence and created pathways to pursue other opportunities.

### Connections and Support Through Lived Experience Participation

#### Profound Understanding From Others With Similar Lived Experiences

Some participants found genuine understanding and connection through shared lived experiences. Lenny (consumer) previously found explaining their mental health to others both, effortful and taxing. However, Lenny’s interactions with other team members were much more natural and comfortable as they bonded over shared experiences:

I didn’t necessarily want to have to explain my situation to my friends, and I felt comfortable sharing my experience with other consumers and carers. (Lenny, consumer)

Similarly, Jordan (carer) had experiences where others didn’t “get it.” Jordan thought their journey of being a carer would remain within themselves until they had the opportunity to interact with other carers. Jordan described the feeling of being understood as a meaningful benefit to participating in lived experience opportunities.

#### Carers Felt Understood and Supported Through Engagement

Connection and support from others were a recurring theme for carers. Often, carers felt unsupported and alone in the journey of navigating the system and caring for their loved ones. Julia (carer) mentioned they were “floundering on my own” and “didn’t feel support from anywhere.”

With lived experience engagement, carers connected and interacted with other carers and coped with loneliness. Jordan (carer) learned more about available services and received practical guidance for them and their loved ones, and Jamie (carer) described LEAT as a “support group.” They appreciated learning about how others cope and exchanged support and wisdom with other team members.

### Considerations for Supporting Consumers and Carers in a Team

#### Importance of Having Diverse Voices

Having diverse lived experiences, identities, and backgrounds in the team was important for most participants. Jamie (carer) recognized the subjectivity of lived experience and how it was just “one point of view” instead of a “gold standard.” Charlie (consumer) believed that the sharing of “different wisdom” contributed to better understanding of issues, which led to better decisions. Lenny (consumer) and Jordan (carer) also respected differences and believed it was necessary to incorporate “fresh blood” and people with “recent experience” to reflect the current state of the mental health system.

### Diverse Views on Working With Both Consumers and Carers

The LEAT consists of both consumers and carers. Although none of the participants were strongly against having a combined team, their level of acceptance toward this structure differed.

Vicki (consumer), Julia (carer), and Jordan (carer) valued others’ perspectives and believed they could “learn” and empathize with others. Charlie (consumer) enjoyed being on the same team with carers. They found a sense of warmth and familiarity when listening to carers’ perspectives:

It makes me smile quite a lot . . . it’s like having my (carer) stay on the chat with me. (Charlie, consumer)

On the contrary, Jamie (carer) initially had doubts about having carers and consumers on the same team, due to their differing perspectives and the “conflict between carers and consumers.” After the experience of working in LEAT, Jamie was somewhat reassured and attributed the “reasonably well” outcome to the deliberate selection of subjects designated to the team.

Lenny (consumer) observed that having a team of both consumers and carers had started to become the norm in the sector.

#### Qualities of Supportive Facilitators

Most participants pointed out the significant role of facilitators in creating and maintaining a safe and inviting environment. Supportive facilitators should be encouraging while also respecting participants’ autonomy. When Charlie (consumer) shared their experiences, they felt accepted and not judged. Similarly, Lenny (consumer) described feeling comfortable sharing their journey but noted how facilitators also respected participants’ boundaries when they chose not to share.

Facilitators should also be approachable and check in with participants regularly. Jordan (carer) felt confident in approaching facilitators if they had any uncomfortable experiences. They trusted the facilitators enough to be honest with them, and they were assured their feedback would be given genuine attention. Vicki (consumer) added that it was helpful when facilitators were observant and noticed when participants were struggling. A sincere check-in could be impactful and valuable.

Julia commented on how body language can also create a comfortable dynamic. They praised the facilitator’s social awareness and body language.

(Facilitator) just knew when to come in, when to stand back, when to smile. (Julia, carer)

### Considerations for Setting Up Voluntary Lived Experience Participation Roles

#### Cost and Constraints of Lived Experience Participation

While their experiences were predominantly positive, Julia (carer) and Jordan (carer) acknowledged the cost and constraints of lived experience participation. Julia (carer) noted that sharing experiences could involve reliving traumatic moments, highlighting the need for increased personal support:

I felt shocking for days after that. . .it takes you into that zone where you’re really living the moment, which is sometimes pretty awful, and like the emotional level, it’s pretty intense, and there should have been more support. (Julia, carer)

Jordan (carer) also highlighted how lived experience opportunities can be time and energy-consuming. Consumers and carers cannot always participate due to competing demands.

#### A Need for Increased Accessibility

Participants mentioned that despite recent improvements, there was still a need for lived experience opportunities to be more accessible. Jordan (carer) and Julia (carer) expressed that had they not been invited; they would not have looked for opportunities themselves. However, Vicki (consumer) felt it was crucial for consumers to have autonomy over their choice of engagements instead of getting “cherry-picked.”

#### Differing Attitudes on Paid Roles

All participants commented on the remuneration of lived experience engagements, but there were differences in the importance of payment. Vicki (consumer) considered receiving payment for lived experience engagements as significant progress in the field. They believed consumers’ and carers’ work was of equal value to other staff members and, therefore, should be paid:

I’m not of lesser value. I am hardworking. I get paid for my time. Therefore, I am valued. (Vicki, consumer)

Julia (carer) expected remuneration when the participation opportunities demanded more of their experiences or skills. They also noted that the payment acknowledged the value of lived experience.

For Charlie (consumer) and Jordan (carer), although payment was not a motivation to engage in lived experience opportunities, they found it “nice” and “useful” in managing their finances and daily expenses. Likewise, Lenny (consumer) stated that payment was not a motivation and was of less importance to them than to others. For Jamie (carer), payment was not necessary, but they respected the standard.

## Discussion

Consumers and carers ascribed various meanings to their lived experience engagements in mental health services. As such, lived experience participation can be considered a human occupation as it involves doing activities (e.g., attending meetings) with meaning (e.g., personal growth) in the context of one’s life, health condition, and the world (e.g., being a consumer in the mental health sector; White et al., 2019). Wilcock’s (1998, 2006) framework can be used to further understand the meaning of engagement beyond “doing” by exploring the dimensions of “being”, “becoming,” and “belonging.”

In this study, the dimension of “doing,” the occupation, is participation in lived experience opportunities. The dimensions “being,” “becoming,” and “belonging” are then connected to the meanings participants ascribed to “doing.”

“Being” is embracing one’s own distinctiveness and unique contributions. To “be” is to rediscover oneself frequently in an accepting way (Hammell, 2004). Participants shared their experiences of loneliness, trauma, and dissatisfaction with the mental health system. Through their engagements, participants could redefine their journeys, and attribute positive meanings to their lived experiences. For consumers and carers in this study and in literature, one of the strongest motivators to join lived experience roles was to share their learned wisdom from the past and facilitate change in others’ lives or the mental health system (Cox et al., 2021; Kessing, 2022). This is an embodiment of “being,” embracing being a consumer or a carer, and the rediscovery of latent abilities in addition to redefining negative experiences.

“Becoming” holds the notion of envisioning future selves, growth, and embracing new possibilities (Hammell, 2004). To date, there has been limited literature focusing on the self-development of consumers and carers who participate in lived experience opportunities. Participants in this study found that their engagements brought about empowerment and growth. Both consumers and carers agreed their roles provided routines and structure and allowed them to occupy time meaningfully. But “becoming” has substantial meaning for consumers particularly, especially for those facing employment challenges. The results from Waghorn et al. (2012) found that consumers diagnosed with psychotic disorders reported multiple individual and systemic barriers to employment. Not only did lived experience participation expand consumers’ skills and confidence, but it also showed consumers the value of their lived experiences. In this sense, becoming is manifested through the opportunities and developments associated with lived experience participation. Participants were building on their roles as consumers and carers; using their skills and confidence to envision different possibilities in life.

“Belonging” refers to connecting with others who share similarities through engagement in meaningful occupations (Hitch et al., 2014). All participants established profound connections with other consumers and carers through their lived experience participation. These social connections were unique as they were formed through people’s shared lived experiences. This meaning resonates especially strongly with carers. Carer participants expressed their previous struggles with “belonging”—how they felt alone and unsupported, similar to the findings of Goodwin and Happell (2007), where carers expressed constant feelings of isolation and exclusion. However, by participating in lived experience opportunities, participants in this study were able to share, understand and connect with carers and consumers with similar experiences.

In addition to understanding the meanings, this study also illuminated the personal impact of lived experience engagement. Participants acknowledged that sharing lived experience can be challenging, as it involves reliving traumatic moments and may lead to increased vulnerability or sensitivity. This observation is consistent with the literature (Meehan & Glover, 2007), where consumers reported feeling over-exposed after sharing their honest selves. In practice, safe-enough and intentional approaches are needed to protect participants’ emotional well-being while sharing their experiences (Gee et al., 2016; Hawgood et al., 2022).

Paid remuneration was another meaningful impact of lived experience participation. All participants reported a consistent remuneration experience, which contrasts with the findings of Ehrlich et al. (2020) and McDonald and Szczepanska (2021), where participants reported inconsistent remuneration. Most participants expressed that while paid remuneration was not their main motivation, it was still meaningful and important to them. Remuneration was particularly meaningful for consumers in this study as it reduced the impacts of being constantly unemployed and underemployed, and created a pathway of opportunities. Most importantly, the payments acted as recognition and acknowledgment of their contributions and equal value. This is consistent with the expectations of people with lived experience (Sartor, 2023) and WHO (2023) recommendations.

One of the study’s unique findings was the significance of establishing a safe-enough environment. This term reflects a person’s individual and subjective perception of having sufficient safety to participate (Reynolds, 2012). Participants had overwhelmingly positive experiences in lived experience participation, contrary to the existing literature. Previous evidence indicated that consumers often faced negative attitudes from health professionals (Gee et al., 2016), carers’ voices were frequently disregarded (Goodwin & Happell, 2007), and consumers’ and carers’ inputs were not exerting meaningful influence due to tokenism (Kessing, 2022). By contrast, participants in this study found their lived experience participation journey to be satisfying, rewarding, and validating; and were confident they were making changes to mental health services and systems. Participants appreciated having supportive facilitators who created a safe-enough space. Other than the qualities listed in the literature such as respect, trust, listening, and acknowledgment (Ehrlich et al., 2020); participants identified additional qualities of supportive facilitators: being approachable, respecting participants’ autonomy, and regularly checking in.

Occupational therapists in the mental health sector should consider engaging with consumers and carers in service design and development in addition to collaborating with the lived experience workforce, which is a recognized competency in the *Mental health occupational therapy capability framework* (Occupational Therapy Australia, 2025). With expertise in the therapeutic nature of “doing,” occupational therapists are well-positioned to support lived experience engagements. The occupational therapy curriculum should also equip emerging occupational therapists to support meaningful participation in lived experience opportunities within safe-enough environments.

Another takeaway is the necessity of incorporating diverse voices. Participants highlighted that lived experiences are subjective and the inclusion of varied and recent experiences allows a more comprehensive overview of the mental health system. Daya et al. (2020) also emphasized the importance of including diverse and challenging views to properly represent the heterogeneity of consumer populations.

Finally, this study briefly explored the perspective of working in a team with both consumers and carers. Participants in this study valued the learnings and insights this structure provided. However, they also acknowledged that conflict sometimes exists between consumers and carers. This finding is in line with Roennfeldt et al.’s (2023) study, where consumer and caregiver participants from the lived experience workforce identified differences in priorities, perspectives, and practices. In this study, participants credited the success of having a team with both consumers and carers to the safe-enough environment. The facilitators actively considered power dynamics and involved people with relevant lived experience, who were most impacted by decisions.

### Limitations

The study’s methodological limitations should be considered when interpreting the findings. It should be acknowledged that details of participation opportunities were omitted to protect confidentiality, limiting the exploration of the relationship between participants’ experiences and specific types of engagement.

In addition, self-selection bias may also influence the trustworthiness of this study. It was possible that consumers and carers who had positive experiences were more inclined to join LEAT and volunteer to be interviewed. As a result, people with less favorable opinions may be underrepresented.

### Future Research

This study focused on exploring the meaning and impact of people’s participation in voluntary, advisory lived experience roles in a community mental health setting. Future research could focus on consumers’ and carers’ perspectives of lived experience opportunities in other settings (e.g., public health). Moreover, including participants who chose to discontinue their participation may offer insight into the reasons someone chooses to participate or not in lived experience opportunities. In terms of understanding meaning, this study adopted the doing, being, becoming, belonging framework. Exploring other ways of understanding personal meaning could provide alternate perspectives.

## Conclusion

This study highlights how lived experience engagement is meaningful to consumers and carers in different ways. Through their engagements in service design, development and research, participants redefined negative experiences into positive contributions; had opportunities for self-growth; and fostered connections. The findings provide practical guidance based on the perspectives of consumers and carers to create opportunities and maximize the benefits of lived experience engagement in mental health service design and development. This involves incorporating diverse opinions, acknowledging the demands of sharing lived experiences, having supportive facilitators, and providing paid remuneration.
